# Mitochondrial Dysfunction in Diabetic Neuropathy: An Evidence Map of Molecular Mechanisms, Translational Biomarkers, and Emerging Implications for Oral Neuropathy—A Scoping Review

**DOI:** 10.3390/ijms27198647

**Published:** 2026-09-27

**Authors:** Kurnia Hayati Rahman, Ujjal K. Bhawal, Jian Wang, Junmin Wang, Kristanti Parisihni

**Affiliations:** 1 Department of Oral Medicine, Faculty of Dentistry, Universitas Hang Tuah, Surabaya 60111, Indonesia; 2 Department of Physiology, Saveetha Medical College and Hospital, Saveetha Institute of Medical and Technical Sciences, Chennai 600077, Tamil Nadu, India; 3 Research Institute of Oral Science, School of Dentistry at Matsudo, Nihon University, Chiba 271-8587, Japan; 4 Nanozyme Laboratory in Zhongyuan, Aging Decoding and Regeneration Institute, School of Basic Medical Sciences, State Key Laboratory of Antiviral Drugs, Pingyuan Laboratory, Zhengzhou University, Zhengzhou 450001, China; jianwang2020@outlook.com; 5 Department of Human Anatomy, School of Basic Medical Sciences, Zhengzhou University, Zhengzhou 450001, China; wangjunmin@zzu.edu.cn; 6 Department of Oral Biology, Faculty of Dentistry, Universitas Hang Tuah, Surabaya 60111, Indonesia; kristanti.parisihni@hangtuah.ac.id

**Keywords:** diabetic peripheral neuropathy, mitochondrial dysfunction, oxidative stress, mitochondrial homeostasis, mitophagy, Schwann cells, biomarkers, oral neuropathy, burning mouth syndrome, scoping review

## Abstract

Diabetic peripheral neuropathy (DPN) is a common and disabling complication of diabetes, but treatment still focuses largely on symptom control rather than modification of the underlying nerve injury. Mitochondrial dysfunction is increasingly implicated in the response of peripheral nerves to chronic hyperglycemia, although the literature is spread across different mitochondrial pathways, experimental models, biomarkers, and clinical settings. We therefore conducted a scoping review, following the Joanna Briggs Institute framework, to map this evidence and examine how far it has extended to oral and craniofacial neuropathic manifestations. PubMed, Scopus, and SpringerLink were searched using a predefined Population–Concept–Context strategy, and two reviewers independently screened records in Rayyan. Of 40,863 records identified, 853 studies met the eligibility criteria; 462 (54.2%) were published between 2021 and 2026. Most studies were preclinical. In the hierarchical evidence map, oxidative stress/redox imbalance was the largest evidence-mapping category (*n* = 591; 69.3%), followed by Schwann-cell degeneration (*n* = 147; 17.2%) and bioenergetic dysfunction (*n* = 77; 9.0%), whereas mitophagy (*n* = 14; 1.6%) and mitochondrial dynamics (*n* = 14; 1.6%) were much less frequently represented. Craniofacial neuropathy (*n* = 6; 0.7%) and oral neuropathy (*n* = 4; 0.5%) represented only a small proportion of the evidence map. Across these domains, recurrent pathways included Nrf2/Keap1 antioxidant defense, NF-κB-associated neuroinflammation, mitochondria-dependent apoptosis, and AMPK-SIRT-PGC-1α signaling. Clinical translation was limited, although mitochondrial DNA copy number, SOD3, and 8-hydroxy-2′-deoxyguanosine were reported as candidate biomarkers. Overall, the expanded evidence base confirms substantial growth in mitochondrial research in diabetic neuropathy but remains predominantly preclinical, with a particularly pronounced translational gap in oral and craniofacial neuropathy.

## 1. Introduction

Diabetes mellitus (DM) is a major global health problem with a wide range of chronic microvascular and neurological complications [1]. Among them, diabetic peripheral neuropathy (DPN) is especially common and clinically important. It can lead to progressive sensory loss, neuropathic pain, impaired peripheral nerve function, and a greater risk of ulceration and amputation [1,2]. These therapeutic limitations have encouraged investigation of interventions targeting underlying mechanisms such as mitochondrial dysfunction, oxidative stress, and neuroinflammation [3].

Mitochondrial dysfunction is increasingly implicated in the pathophysiology of diabetic peripheral neuropathy and has been linked to oxidative stress, neuroinflammation, and neuropathic pain [3]. Sensory neurons and Schwann cells are metabolically demanding cells that rely on oxidative phosphorylation, mitochondrial quality control, and appropriate movement of mitochondria within the cell [4]. Hyperglycemia can disturb each of these processes. The reported changes include increased reactive oxygen species (ROS), reduced ATP production, mitochondrial DNA damage, defective mitophagy, altered mitochondrial dynamics, and activation of inflammatory and cell-death pathways [5]. The abnormalities are closely linked, and thus it is unlikely that any one of them acts alone in the progression of DPN.

This growing mechanistic literature has also encouraged the search for biomarkers and therapeutic targets that are more closely tied to disease biology. Mitochondrial DNA copy number and several oxidative-stress-related molecules have been explored as possible indicators of disease severity or treatment response. At the same time, Nrf2, NF-κB, AMPK, sirtuins, and PGC-1α have emerged repeatedly as regulatory nodes of interest [5,6]. What remains less clear is how strongly each of these areas is represented across preclinical and clinical research, and which have progressed furthest toward clinical translation.

An additional question is whether this mitochondrial framework has relevance to neuropathic symptoms in the oral and craniofacial region. Burning mouth syndrome (BMS), oral dysesthesia, lingual neuropathy, glossodynia, and diabetic trigeminal neuropathy may present with burning pain, altered sensation, or taste disturbance despite the absence of obvious mucosal lesions [7,8]. The oral cavity is extensively innervated by sensory fibers, including trigeminal afferents, and alterations in oral and trigeminal sensory function have been demonstrated in neuropathic oral pain conditions [9]. This makes mitochondrial mechanisms established in peripheral neuropathy a reasonable starting point for investigating diabetes-associated oral sensory dysfunction. It does not, however, mean that the same mechanisms have already been demonstrated in oral neuropathic disorders.

The unresolved question is therefore broader than whether mitochondria are involved in DPN. We also need to know where the evidence is concentrated, which mechanisms have been studied, how much of the work has reached patients, and whether comparable mechanisms have been examined in craniofacial neuropathy. Mapping these differences can help separate well-established areas from those that remain largely hypothetical.

Because this literature spans experimental, translational, and clinical studies with considerable methodological heterogeneity, a scoping review is more appropriate than a pooled effectiveness analysis. It allows the field to be mapped as a whole, while also showing where evidence is abundant and where important gaps remain.

Mitochondria provide a useful framework for integrating several interconnected mechanisms implicated in DPN. In addition to ATP production, they regulate redox balance, calcium homeostasis, mitophagy and cell survival, all of which can affect neuronal and glial function. Mitochondrial dysfunction may therefore represent a converging mechanism linking metabolic stress to progressive peripheral nerve injury.

Diabetes can also affect sensory function in the oral and craniofacial region, with patients experiencing symptoms such as oral burning, dysesthesia, altered sensation, and trigeminal or lingual neuropathic symptoms. These manifestations are heterogeneous, however, and should not be regarded as established oral counterparts of DPN. How they relate to systemic diabetic neuropathy, particularly through mitochondrial mechanisms, remains poorly understood.

The aim of this scoping review was therefore to map research on mitochondrial dysfunction in diabetic neuropathy, describe the main molecular pathways and experimental models, summarize candidate translational biomarkers, and assess how far this mechanistic framework has been investigated in oral and craniofacial neuropathic disorders.

## 2. Material and Methods

### 2.1. Scoping Review Framework

This scoping review was conducted according to the Joanna Briggs Institute (JBI) methodology for scoping reviews described by Peters et al. [10]. The review followed six stages: defining the review question; developing eligibility criteria; designing and conducting the search; selecting studies; extracting data; and analyzing and presenting the evidence. Reporting was guided by the PRISMA extension for Scoping Reviews (PRISMA-ScR) [11].

### 2.2. Review Questions and PCC Framework

The primary question was: What is the current evidence landscape of mitochondrial dysfunction in diabetic neuropathy, and what translational gaps remain for oral neuropathic disorders? The Population–Concept–Context (PCC) framework was used to align eligibility criteria, search strategy, data extraction, and evidence mapping.

1. What mitochondrial mechanisms of diabetic neuropathy have been studied?
2. What experimental models and patient populations have been used to study these mechanisms?
3. What are the most mature molecular pathways and biomarkers for translation?
4. To what extent have mitochondrial mechanisms been investigated in oral and craniofacial neuropathic disorders?

These questions were used prospectively to structure study selection, data charting, and the descriptive evidence map.

### 2.3. Eligibility Criteria

Original research studies involving individuals or experimental models of diabetes-associated neuropathy were eligible, including type 1 or type 2 diabetes, DPN, diabetic small-fiber neuropathy, animal models, and cellular or mechanistic models. Eligible studies investigated mitochondrial dysfunction or closely related processes, including oxidative stress, mitochondrial bioenergetics, mitophagy, mitochondrial dynamics, mitochondrial biogenesis, axonal mitochondrial dysfunction, or Schwann-cell injury. The context included preclinical, translational, and clinical research, with additional consideration of oral and craniofacial neuropathic disorders, including diabetes-associated trigeminal and other craniofacial sensory neuropathies, to identify direct evidence and translational gaps.

Only full-text articles published in English were included. Review articles, conference abstracts, editorials, letters, case reports, case series, and studies focused exclusively on diabetic complications other than neuropathy without investigation of mitochondrial dysfunction were excluded.

### 2.4. Search Strategy

PubMed, Scopus, and SpringerLink were searched for studies published from January 2010 through August 2026. Five sequential search sets were designed to capture: (1) core evidence on mitochondrial dysfunction in diabetic neuropathy; (2) mechanistic evidence from experimental models; (3) mitochondrial dysfunction in oral and craniofacial neuropathic disorders; (4) translational evidence linking diabetes-related neuropathy with oral neuropathic manifestations; and (5) evidence directly integrating diabetes, mitochondrial dysfunction, and oral neuropathy (Table 1). Controlled vocabulary, including Medical Subject Headings where applicable, and free-text terms were combined with Boolean operators and adapted to each database. Following re-evaluation of the search coverage, the searches were repeated for the period from January 2010 through August 2026 to ensure complete retrieval and screening of eligible records within this period. Complete database-specific strategies are provided in Supplementary Table S1.

Search Set Focus Purpose Search Set 1 Diabetic neuropathy + mitochondrial dysfunction was used to identify the core body of evidence on mitochondrial mechanisms in diabetic neuropathy. Search Set 2 Experimental models was used to retrieve mechanistic evidence from animal and cellular studies. Search Set 3 Mitochondrial Dysfunction and Oral Neuropathic Diseases was used to review the literature on mitochondrial dysfunction in neuropathic disorders of the oral and craniofacial region. Search Set 4 Diabetes-related neuropathy + oral neuropathic disorders was used to identify translational evidence linking diabetic neuropathy with oral neuropathic manifestations. Search Set 5 Diabetes + mitochondrial dysfunction + oral neuropathic disorders was used to identify integrated evidence directly connecting all three concepts and reveal potential knowledge gaps.

This staged strategy was intended to map the evidence continuum from established mitochondrial mechanisms in DPN to direct oral and craniofacial evidence, allowing the absence of translation to be identified explicitly rather than inferred.

### 2.5. Study Selection

All records retrieved from the updated searches were imported into EndNote and subsequently uploaded to Rayyan for deduplication and screening. Duplicate records identified in Rayyan were removed before title and abstract screening. Rayyan’s AI-assisted functions were used only to support screening efficiency and organization; all eligibility decisions were made independently by human reviewers. Two reviewers independently screened titles and abstracts against the predefined PCC-based criteria and assessed potentially eligible full texts. During independent screening, Rayyan’s blind mode was enabled so that each reviewer was unaware of the other reviewer’s decisions until the screening process was complete [12]. Disagreements were resolved by discussion or consultation with a third reviewer. The selection process was documented using a PRISMA-ScR flow diagram.

### 2.6. Data Charting

A standardized data-charting form was developed and refined to ensure consistency. Extracted variables included publication characteristics, study design, biological model or clinical population, neuropathy phenotype, mitochondrial mechanism, major molecular pathway or biomarker, and intervention type. Oral and craniofacial neuropathic manifestations were charted separately. Craniofacial manifestations included trigeminal and other diabetes-associated craniofacial sensory neuropathies, whereas oral manifestations included BMS-like symptoms, diagnosed BMS, oral dysesthesia, lingual neuropathy, glossodynia, and other oral sensory disturbances.

### 2.7. Evidence Synthesis

Data were synthesized descriptively in accordance with scoping-review methodology. Studies were summarized by publication period, study design, model or population, neuropathy phenotype, mitochondrial mechanism, and intervention type. To avoid double counting of studies reporting multiple mechanisms, each study was assigned to a single principal evidence-mapping category using a hierarchical classification framework based on the most upstream and biologically specific feature identified. Priority was applied sequentially to oral neuropathy, craniofacial neuropathy, Schwann-cell degeneration, mitochondrial dynamics, mitophagy, bioenergetic dysfunction, and oxidative stress. This hierarchy was used solely for evidence-mapping purposes and does not imply a ranking of biological importance. Priority was given first to the review-specific translational domain (oral and craniofacial neuropathy), followed by cell-type-specific and more mechanistically defined mitochondrial processes, whereas broader and overlapping features such as oxidative/redox stress were assigned lower priority to minimize double counting. Accordingly, studies meeting the criteria for the review-specific oral or craniofacial domains, or demonstrating a more specific mitochondrial or cell-type-related process, were assigned preferentially to the corresponding category; studies not meeting the criteria for a higher-priority category were classified under oxidative stress/redox imbalance. The oxidative-stress category encompassed evidence of oxidative/redox imbalance and associated antioxidant-defense pathways, including Nrf2/Keap1-related signaling, while recognizing substantial biological overlap with inflammatory and apoptotic pathways. Additional processes, including mitochondrial DNA abnormalities, mitochondrial biogenesis, axonal mitochondrial dysfunction and transport, inflammatory signaling, and cell-death pathways, were characterized within the relevant principal category rather than treated as additional mutually exclusive quantitative categories. Where clinical data were available, mitochondria-related biomarkers and their reported associations with neuropathy severity, nerve function, or treatment response were summarized. Direct evidence concerning oral neuropathic manifestations was mapped separately.

### 2.8. Critical Appraisal

Formal risk-of-bias or methodological quality appraisal was not undertaken because the purpose of this scoping review was to map the extent, characteristics, and gaps of a heterogeneous evidence base rather than to estimate intervention effectiveness. This decision should be considered when interpreting mechanistic and translational conclusions.

## 3. Results

Search Sets 1 and 2 yielded a great majority of eligible studies, confirming that the literature is concentrated on mitochondrial mechanisms in conventional diabetic neuropathy and experimental models.

### 3.1. Study Selection

The updated database search identified 40,863 records: 30,476 from PubMed, 10,314 from Scopus, and 73 from SpringerLink. After removal of 11,611 duplicate records, 29,252 unique records underwent title and abstract screening. Of these, 27,791 records were excluded, including 5689 review articles, 141 case reports, and 21,961 records unrelated to the core review concepts. A total of 1461 full-text articles were assessed for eligibility, of which 608 were excluded. Ultimately, 853 studies were included in the review (Figure 1).

### 3.2. Characteristics of the Included Studies

A total of 853 studies met the eligibility criteria. Of these, 391 (45.8%) were published between 2010 and 2020 and 462 (54.2%) between 2021 and 2026 (Table 2). When publication trends were examined by calendar year, 532 studies (62.4%) were published between 2020 and 2026, compared with 321 (37.6%) between 2010 and 2019 (Figure 2). Publication activity increased substantially after 2020 and peaked at 103 publications in 2025. The lower count in 2026 reflects the partial-year search period (January–August 2026) and should not be interpreted as an annual decline. The evidence base remained predominantly preclinical: animal studies accounted for 415 (48.7%) studies, combined animal and in vitro studies for 232 (27.2%), and in vitro studies for 39 (4.6%), whereas 167 (19.6%) were clinical studies. Diabetic neuropathy was the most frequent neuropathy phenotype (*n* = 551; 64.6%), followed by painful DPN (*n* = 248; 29.1%), cardiovascular autonomic neuropathy (*n* = 31; 3.6%), craniofacial neuropathy (*n* = 19; 2.2%), and oral neuropathy (*n* = 4; 0.5%).

### 3.3. Evidence Map of Mitochondrial Dysfunction in Diabetic Neuropathy

Hierarchical evidence mapping of the 853 included studies showed that oxidative stress/redox imbalance was the predominant category, comprising 591 studies (69.3%). Schwann-cell degeneration was the second most frequent category (*n* = 147; 17.2%), followed by bioenergetic dysfunction (*n* = 77; 9.0%). In contrast, relatively few studies were assigned to mitophagy (*n* = 14; 1.6%) or mitochondrial dynamics (*n* = 14; 1.6%). Studies principally assigned to craniofacial neuropathy (*n* = 6; 0.7%) and oral neuropathy (*n* = 4; 0.5%) were particularly limited (Table 3; Figure 3).

Regarding intervention status, 588 studies (68.9%) investigated pharmacological interventions, 34 (4.0%) investigated non-pharmacological interventions, and 231 (27.1%) did not evaluate an intervention. Because many studies investigated multiple interconnected mechanisms, each study was assigned to a single principal category according to the hierarchical framework to avoid double counting. Thus, the quantitative distribution reflects principal evidence-mapping classification rather than mutually exclusive biological activity; additional mitochondrial, inflammatory, oxidative, and cell-death pathways could coexist within individual studies. The complete list of included studies is provided in Supplementary Table S2.

The distribution of principal evidence categories by study design and intervention type is summarized in Figure 3.

The volume of publications on mitochondrial dysfunction in diabetic neuropathy grew significantly post-2020, peaking in 2025 with 103 publications. Note: The smaller number for 2026 is due to the partial year search period (January–August 2026) and not a decrease year-on-year. Of the total studies, 532 (62.4%) were published between 2020 and 2026, and 321 (37.6%) studies were published between 2010 and 2019.

Major molecular pathways and representative markers associated with each mitochondrial domain are summarized in Supplementary Table S3.

Overall, the evidence map identifies a rapidly expanding but uneven research landscape. Oxidative stress/redox imbalance was the largest principal evidence category, whereas bioenergetic dysfunction was less frequently represented. Mitochondrial dynamics and mitophagy were underrepresented in comparison, with limited clinical translation. The near absence of direct oral or craniofacial mitochondrial studies represents the most pronounced translational gap.

Studies are distributed according to principal evidence category, study design, and intervention type. To avoid double counting, studies reporting multiple mechanisms were assigned to a single principal evidence category using the hierarchical classification framework. Studies not meeting the criteria for a higher-priority evidence category were classified under oxidative stress/redox imbalance. Percentages for study design and intervention were calculated within each evidence category.

## 4. Discussion

### 4.1. An Uneven but Rapidly Expanding Mitochondrial Evidence Landscape

This scoping review mapped 853 studies published between 2010 and August 2026 examining mitochondrial dysfunction and related processes in diabetic neuropathy. The most frequently represented principal evidence category was oxidative stress/redox imbalance (*n* = 591; 69.3%), followed by Schwann-cell degeneration (*n* = 147; 17.2%) and bioenergetic dysfunction (*n* = 77; 9.0%). Conversely, mitophagy (*n* = 14; 1.6%) and mitochondrial dynamics (*n* = 14; 1.6%) were underrepresented. There was notably limited evidence directly mapped to craniofacial neuropathy (*n* = 6; 0.7%) and oral neuropathy (*n* = 4; 0.5%). These distributions reflect the relative representation of research themes within the literature and should not be interpreted as measures of mechanistic importance, evidence strength, or therapeutic validation.

The revised evidence map therefore reinforces a marked imbalance between the extensive literature on conventional diabetic neuropathy and the limited evidence addressing craniofacial and oral neuropathic manifestations. Although mitochondrial mechanisms identified in peripheral diabetic neuropathy provide a biologically plausible framework for investigating these manifestations, they cannot be assumed to operate identically in trigeminal or oral sensory tissues without direct experimental or clinical validation.

Taken together, the literature portrays DPN as a network of interacting metabolic and cellular disturbances. Chronic hyperglycemia disrupts redox balance, mitochondrial energy production, organelle quality control, inflammatory signaling and cell survival. These processes may also reinforce each other [13]. For example, oxidative injury can damage the electron transport chain and mitochondrial DNA; damaged mitochondria can then generate further ROS; and inadequate mitophagy may allow dysfunctional organelles to persist, prolonging neuronal and glial stress [13].

This interaction is particularly meaningful in peripheral nerves. Long axons require a continuous supply of ATP and effective mitochondrial trafficking to distal terminals [14]. Schwann cells are essential for myelin maintenance, metabolic support of axons, and peripheral nerve repair [15]. The substantial representation of Schwann-cell degeneration in the revised evidence map highlights the considerable research attention devoted to glial pathology within the broader DPN literature. There are also signs that the field is moving beyond descriptive mechanisms toward intervention studies and biomarker development. That transition, however, is still incomplete. Experimental pharmacological studies are plentiful, whereas validated mitochondrial biomarkers and mechanism-based treatments suitable for routine clinical use remain scarce.

### 4.2. Oxidative/Redox Stress as a Dominant Molecular Hub

Oxidative stress was the largest category in the hierarchical evidence map, accounting for approximately 69% of the included studies. This figure needs to be interpreted considering the classification method. Studies with more specific evidence of mitochondrial dynamics, mitophagy, bioenergetic dysfunction, Schwann-cell degeneration, or oral neuropathy were assigned to those categories first; oxidative stress therefore functioned as a broader redox-centered category for the remaining studies. Accordingly, the 591 studies should not be interpreted as 591 direct measurements of mitochondrial ROS. Biologically, however, redox disturbance remains highly relevant to DPN. Chronic hyperglycemia can increase oxidative stress through the polyol pathway, protein kinase C activation, the hexosamine pathway, and AGE-RAGE signaling [1]. Mitochondrial dysfunction in peripheral neuropathy is associated with impaired mitochondrial membrane potential and alterations in respiratory-chain components, which can compromise cellular bioenergetics [16]. Once mitochondria are injured, they may generate still more ROS, creating a self-reinforcing cycle of oxidative damage.

Nrf2/Keap1/ARE signaling was recurrently represented within the redox-centered literature and is an important antioxidant pathway implicated in diabetic neuropathy [17]. Several experimental interventions were linked to Nrf2-associated antioxidant signaling, although the specific pathways differed among compounds. Deguelin was reported to ameliorate experimental diabetic neuropathy through Nrf2 signaling [18], whereas oltipraz reduced high-glucose-induced oxidative stress and apoptosis in Schwann cells through the Nrf2/NQO1 pathway [19]. Hedysarum polysaccharide was similarly associated with modulation of the Keap1/Nrf2 pathway [20]. Other antioxidant or neuroprotective interventions acted through distinct signaling pathways; for example, the curcumin derivative J147 was investigated in relation to AMPK signaling [21]. Downstream antioxidant molecules, including NQO1 and other redox-defense markers, were also evaluated across these studies [19,20,22]. The relevance of this pathway is not confined to DPN; experimental work in other neurological settings has also linked NRF2/HO-1 activation with lower oxidative stress, reduced inflammatory signaling, and changes in glial activation [23].

Apoptotic signaling was also evident in Schwann-cell injury, with decreased Bcl-2, increased Bax, and activation of caspase-3 demonstrated in experimental diabetic neuropathy [24]. Endoplasmic-reticulum stress pathways involving GRP78, caspase-12, and PERK/ATF4/CHOP have been associated with apoptosis and sciatic-nerve injury in experimental DPN [25,26].

Neuroinflammatory signaling was another recurrent feature of mapped literature. NF-κB and NLRP3 inflammasome activation were associated with increased proinflammatory cytokine expression, oxidative stress, and mitochondrial dysfunction in experimental diabetic neuropathy [27]. However, modulation of these molecular markers should not by itself be interpreted as evidence that the corresponding pathways are causally required for the neuropathic phenotype. For broader biological context, similar interactions among oxidative stress, inflammation, and neuronal injury have been reported in experimental models of other neurological disorders [28]; however, such evidence was not considered direct evidence of mitochondrial mechanisms in DPN.

Interpretation of intervention studies also requires caution because improvements in oxidative or inflammatory markers may accompany changes in glycemic control or broader metabolic effects and therefore cannot always be attributed specifically to direct mitochondrial actions. Moreover, improvement in molecular markers does not necessarily correspond to improvement in neuropathic symptoms or functional outcomes.

Viewed together, these findings place oxidative/redox stress at an important intersection of antioxidant failure, inflammatory signaling, mitochondria-associated cell death, and other cellular stress responses.

At the same time, the size of this category should not be taken as evidence that oxidative stress represents the strongest or primary causal mitochondrial mechanism in DPN. Its numerical dominance reflects both the large amount of redox-focused research and the hierarchical classification used in this review. Compared with ROS-related injury, mitochondrial turnover, trafficking, cell-specific vulnerability, and clinically useful biomarkers have received much less attention.

### 4.3. Mitochondrial Homeostasis: Dynamics, Mitophagy, and Bioenergetics

Besides redox dysregulation, impaired bioenergetics, defective mitophagy and altered mitochondrial dynamics were identified as tightly intertwined aspects of mitochondrial homeostasis in the evidence map. Chronic hyperglycemia can promote oxidative stress and mitochondrial dysfunction in cells of peripheral nerves, for example, by increasing ROS production and decreasing mitochondrial membrane potential [29]. If quality-control mechanisms fail at the same time, damaged mitochondria may accumulate and further compromise neuronal and Schwann-cell function.

AMPK appeared repeatedly across these domains as an upstream energy sensor linked to PGC-1α, sirtuins, and mitochondrial adaptation. Experimental studies have implicated AMPK-associated signaling in mitochondrial protection and quality control in DPN. For example, α-lipoic acid activated AMPK in experimental DPN [30], whereas the AMPK activator AICAR was reported to prevent and reverse experimental diabetic polyneuropathy in association with regulation of mitophagy [31]. Collectively, these studies suggest that AMPK-SIRT-PGC-1α-related signaling may connect mitochondrial bioenergetics, biogenesis, and quality control; however, the available evidence remains predominantly preclinical and does not establish a universally validated therapeutic target.

Within the bioenergetic literature, changes in AMPK/PGC-1α-related signaling were reported alongside markers of mitochondrial biogenesis, including NRF1, whereas SIRT1- and SIRT3-related pathways were investigated in relation to mitochondrial function, oxidative capacity, and NAD+-dependent metabolic regulation [31,32,33]. These observations provide a rationale for strategies to restore mitochondrial energy metabolism, but evidence is still mostly preclinical.

Mitophagy was comparatively underrepresented in the evidence map. Experimental DPN studies nevertheless implicated mitochondrial quality-control pathways in the handling of damaged mitochondria. AICAR-mediated AMPK activation was associated with regulation of mitophagy in experimental diabetic polyneuropathy [31], while piceatannol was reported to promote mitophagy and mitochondrial biogenesis in experimental DPN and hyperglycemia-induced neurotoxicity [34]. These findings support an association between metabolic sensing and mitochondrial quality control, although pathway-marker changes should be distinguished from experiments demonstrating causal dependency through genetic or pharmacological manipulation.

Mitochondrial dynamics and trafficking were among the least developed areas in the evidence map. In some experimental settings, altered MFN2-related fusion, mitochondrial fragmentation, and impaired axonal mitochondrial transport have been reported in association with diabetic nerve injury [16]. These directional alterations should not be interpreted as universal findings across all DPN models or clinical settings. The relative neglect of these processes is surprising considering that distal axons depend on correctly positioned mitochondria for local energy supply.

These findings are better viewed as parts of one homeostatic system than as separate pathways. Bioenergetics, biogenesis, mitophagy, fusion-fission balance, and mitochondrial trafficking are interdependent, so failure in one process can place additional strain on the others. This may help explain the recurring interest in shared regulators such as AMPK, sirtuins, and PGC-1α.

### 4.4. Schwann-Cell Vulnerability as a Critical Cellular Interface

Schwann cells represent an important cellular interface between metabolic stress and structural nerve damage [35]. They maintain myelin, provide metabolic support to axons, and participate in peripheral nerve repair [35]. In the revised hierarchical evidence map, 147 studies (17.2%) were assigned principally to Schwann-cell degeneration, making it the second most frequently represented category after oxidative stress/redox imbalance. This distribution indicates substantial research attention to Schwann-cell-related pathology, although it does not by itself establish the relative mechanistic importance of Schwann-cell dysfunction in DPN.

The reported Schwann-cell abnormalities were heterogeneous. In the included studies, ZBTB16 was reported to inhibit Schwann-cell dedifferentiation and myelin damage and improve mitochondrial function in experimental DPN [36]. Mechanistically, ZBTB16 transcriptionally activated the mitochondrial regulator PTCD1, and PTCD1 knockdown abolished the protective effects of ZBTB16 on Schwann-cell differentiation and mitochondrial function, providing evidence of pathway dependency [36].

Collectively, these findings position Schwann cells at the intersection of oxidative stress, altered neurotrophic signaling, mitochondrial dysfunction, and cell-death pathways in experimental DPN. However, much of the evidence is based on changes in molecular markers or responses to pharmacological interventions rather than direct demonstrations of pathway dependency. Further cell-specific studies using inhibition, genetic manipulation, or rescue approaches are needed to determine which Schwann-cell abnormalities are causal, reversible, and therapeutically actionable.

### 4.5. Translational Progress: From Mechanisms to Clinical Biomarkers

The predominance of preclinical studies highlights the continuing translational gap in mitochondrial research on DPN. Measurements of mitochondrial respiration, dynamics, and quality control remain concentrated largely in experimental models, whereas clinical studies more commonly evaluate circulating, biochemical, or other surrogate markers. Several candidate biomarkers nevertheless provide preliminary links between mitochondrial or oxidative biology and clinical neuropathy phenotypes.

Among the clinical studies, peripheral-blood mitochondrial DNA copy number (mtDNA-CN) was reported to be reduced in patients with type 2 diabetes and polyneuropathy [5]. Other oxidative-stress-related biomarkers have also been evaluated in clinical DPN studies. Lower serum SOD3 levels were associated with diabetic sensorimotor polyneuropathy and impaired nerve-conduction measures [37], whereas 8-hydroxy-2′-deoxyguanosine (8-OHdG) has been investigated as a marker of oxidative DNA damage in patients with DPN [38]. Other clinical studies evaluated broader oxidative-stress measures; for example, total antioxidant capacity and MDA were assessed in patients with type 2 diabetes with or without DPN [39]. Notably, MDA levels were lower in the DPN group, illustrating that oxidative-stress biomarkers do not necessarily show uniform directional changes across clinical studies. Changes in these markers were sometimes reported alongside changes in neuropathic or functional outcomes; however, such parallel changes do not establish that modulation of oxidative stress mediated the clinical or behavioral response.

Non-coding RNAs have also been investigated as potential clinical biomarkers of DPN. In patients with type 2 diabetes, altered levels of lncRNA FTX and miR-186-5p were associated with DPN [40]. Long non-coding RNAs have also been investigated as potential predictive markers for the early detection of diabetic neuropathy in patients with type 1 diabetes [41]. These findings remain preliminary and require independent validation before their clinical utility can be established.

At present, these candidate biomarkers should be regarded as exploratory rather than clinically validated. Differences in study populations, sample types, assay methods, definitions of neuropathy, and clinical outcomes limit direct comparisons between studies. Moreover, circulating markers may reflect systemic metabolic or oxidative abnormalities associated with diabetes, rather than nerve-specific mitochondrial dysfunction.

Future clinical translation will require prospective studies, standardized assays, neuropathy phenotyping and validation in independent populations. Multimarker approaches combining indices of mitochondrial integrity, oxidative injury, inflammation and metabolic status should be explored, but their clinical utility remains to be determined.

### 4.6. From Peripheral to Oral Neuropathy: A Translational Blind Spot

A major translational gap emerged at the intersection of diabetic neuropathy and oral and craniofacial sensory disorders. In the revised dataset, 19 studies (2.2%) involved a craniofacial neuropathy phenotype and 4 (0.5%) involved an oral neuropathy phenotype. Under the hierarchical evidence-mapping framework, 6 studies (0.7%) were assigned principally to craniofacial neuropathy and 4 (0.5%) to oral neuropathy. Thus, although the expanded search identified a broader craniofacial literature than the original analysis, direct evidence at the oral-mitochondrial interface remained sparse.

Oral evidence also requires careful phenotypic distinction. Studies reporting BMS-like symptoms in patients with diabetes [7,8] should not be interpreted automatically as studies of formally diagnosed primary BMS or as confirmation of a distinct diabetic oral neuropathy. In this review, “BMS-like symptoms” refers to burning-mouth-type symptoms reported in association with diabetes or peripheral neuropathy, whereas “BMS” is reserved for studies explicitly identifying the condition as BMS. “Diabetic oral neuropathy” is used only when an oral neuropathic phenotype is specifically attributed to diabetes within the individual study.

The available evidence therefore does not establish that oral or craniofacial neuropathic disorders in diabetes share the mitochondrial mechanisms characterized in conventional DPN. Rather, the peripheral DPN literature provides a mechanistic framework that can be tested in these anatomically distinct sensory systems. Candidate domains for such investigation include oxidative/redox imbalance, mitochondrial bioenergetics, mitochondrial quality control, and clinically measurable mitochondrial or oxidative biomarkers. Craniofacial evidence should also not be equated with oral evidence. Diabetes-associated alterations involving trigeminal or other craniofacial sensory pathways may provide biological relevance to oral sensory dysfunction, but findings from non-oral craniofacial tissues cannot be assumed to represent oral neuropathy directly.

The oral cavity nevertheless represents an accessible clinical site in which systemic metabolic neuropathy may intersect with trigeminal sensory biology. Future studies should combine clearly defined oral-neuropathy phenotypes with systemic neuropathy assessment and direct mitochondria-related measurements. Such studies could determine whether patients with diabetes-associated oral burning, dysesthesia, or other sensory disturbances exhibit mitochondrial signatures comparable to those reported in conventional DPN. Until such evidence is available, mitochondrial involvement in diabetic oral neuropathy should be regarded as a testable hypothesis rather than an established mechanism.

## 5. Strengths, Limitations, and Research Priorities

This review has several strengths. It applies the JBI scoping-review framework to a large and heterogeneous body of evidence and, following re-evaluation of the search and screening process, maps 853 studies published between January 2010 and August 2026 across preclinical, translational, and clinical settings. The hierarchical evidence-mapping approach allowed studies reporting overlapping mitochondrial and neuropathic features to be assigned to a single principal category, thereby minimizing double counting while highlighting both well-represented and underexplored research domains. The expanded analysis also distinguishes oral from broader craniofacial neuropathic evidence, allowing the translational gap in these areas to be characterized more precisely.

Several limitations should be considered when interpreting the evidence map. Only English-language full-text studies were included, and the evidence remained predominantly preclinical. Considerable heterogeneity in experimental models, interventions, mitochondrial measurements, neuropathy phenotypes, and clinical outcomes precluded quantitative synthesis. In addition, no formal risk-of-bias or certainty-of-evidence assessment was performed, consistent with the descriptive mapping purpose of a scoping review. Consequently, the number of studies assigned to a particular category reflects the frequency with which that topic was represented in the literature and should not be interpreted as a measure of evidence quality, mechanistic importance, causal strength, or therapeutic efficacy.

Causal interpretation is further limited by the design of much of the underlying literature. Many studies reported changes in oxidative, inflammatory, apoptotic, or mitochondrial pathway markers without directly testing pathway dependency through inhibition, genetic manipulation, or rescue experiments. Furthermore, some interventions altered glycemic or broader metabolic parameters together with mitochondrial or neuropathic outcomes, making it difficult to distinguish direct mitochondrial effects from improvements secondary to systemic metabolic control. Molecular or biochemical improvement also did not necessarily imply corresponding improvement in neuropathic symptoms or functional outcomes. These considerations emphasize the need to distinguish pathway association from experimentally demonstrated causal dependency.

The hierarchical classification itself also has limitations. Because each study was assigned to a single principal evidence-mapping category, the approach necessarily simplifies biological overlap among oxidative stress, bioenergetic dysfunction, mitophagy, mitochondrial dynamics, Schwann-cell injury, and neuropathic phenotypes. The predominance of oxidative stress/redox imbalance therefore partly reflects the hierarchical classification framework and should not be interpreted as evidence that oxidative stress is the primary mitochondrial mechanism in DPN. Similarly, the relatively small numbers assigned to mitophagy or mitochondrial dynamics indicate limited representation in the mapped literature rather than evidence that these processes are biologically less important.

Important translational gaps also remain. Although 19 studies involved craniofacial neuropathy phenotypes and 4 involved oral neuropathy phenotypes, only 6 studies were assigned principally to craniofacial neuropathy and 4 to oral neuropathy in the hierarchical evidence map. These counts should not be interpreted as evidence that mitochondrial mechanisms established in conventional DPN have been demonstrated in oral or craniofacial sensory disorders. Future priorities therefore include direct mechanistic studies of mitochondrial function in trigeminal and oral sensory systems; greater investigation of mitochondrial dynamics and mitophagy; cell-specific studies in Schwann cells and sensory neurons; and prospective validation of candidate mitochondrial and oxidative biomarkers using standardized clinical phenotyping and assays.

## 6. Conclusions

This scoping review maps 853 studies published between January 2010 and August 2026 examining mitochondrial dysfunction and related processes in diabetic neuropathy. The evidence base was predominantly preclinical and unevenly distributed across research domains. In the hierarchical evidence map, oxidative stress/redox imbalance was the most frequently represented category (*n* = 591; 69.3%), followed by Schwann-cell degeneration (*n* = 147; 17.2%) and bioenergetic dysfunction (*n* = 77; 9.0%), whereas mitophagy (*n* = 14; 1.6%) and mitochondrial dynamics (*n* = 14; 1.6%) were much less frequently represented. These frequencies describe the distribution of literature and should not be interpreted as measures of mechanistic importance, causal strength, or therapeutic validation.

Across the mapped literature, recurrent molecular themes included Nrf2/Keap1-associated antioxidant defense, NF-κB-associated neuroinflammatory signaling, mitochondria-related apoptosis, and AMPK-SIRT-PGC-1α-associated mitochondrial homeostasis. However, much of this evidence is based on pathway-marker changes and pharmacological interventions rather than direct demonstrations of causal dependency. The clinical translation is also restricted. Candidate biomarkers, such as SOD3, 8-OHdG and peripheral-blood mitochondrial DNA copy number, are worthy of further investigation, but require independent validation and standardized phenotyping and measurement to establish their clinical utility.

The translational gap is particularly evident for oral and craniofacial neuropathy. Although the expanded dataset identified 19 studies involving craniofacial neuropathy phenotypes and 4 involving oral neuropathy phenotypes, only 6 and 4 studies, respectively, were assigned principally to these categories in the hierarchical evidence map. The available evidence does not establish that mitochondrial mechanisms characterized in conventional DPN operate similarly in oral or craniofacial sensory disorders. Direct investigation of mitochondrial function in trigeminal and oral sensory systems is therefore needed to determine whether shared molecular signatures exist.

Overall, this evidence map identifies a substantial but predominantly preclinical literature on mitochondrial dysfunction in diabetic neuropathy while highlighting underexplored areas in mitochondrial quality control, clinical biomarker validation, and oral and craniofacial neuropathy. Future studies combining mechanistic perturbation approaches, cell- and tissue-specific mitochondrial measurements, and well-defined clinical phenotypes will be important for determining which mitochondrial abnormalities represent causal and clinically actionable features of diabetic neuropathy (Figure 4).

## Figures and Tables

**Figure 1:**
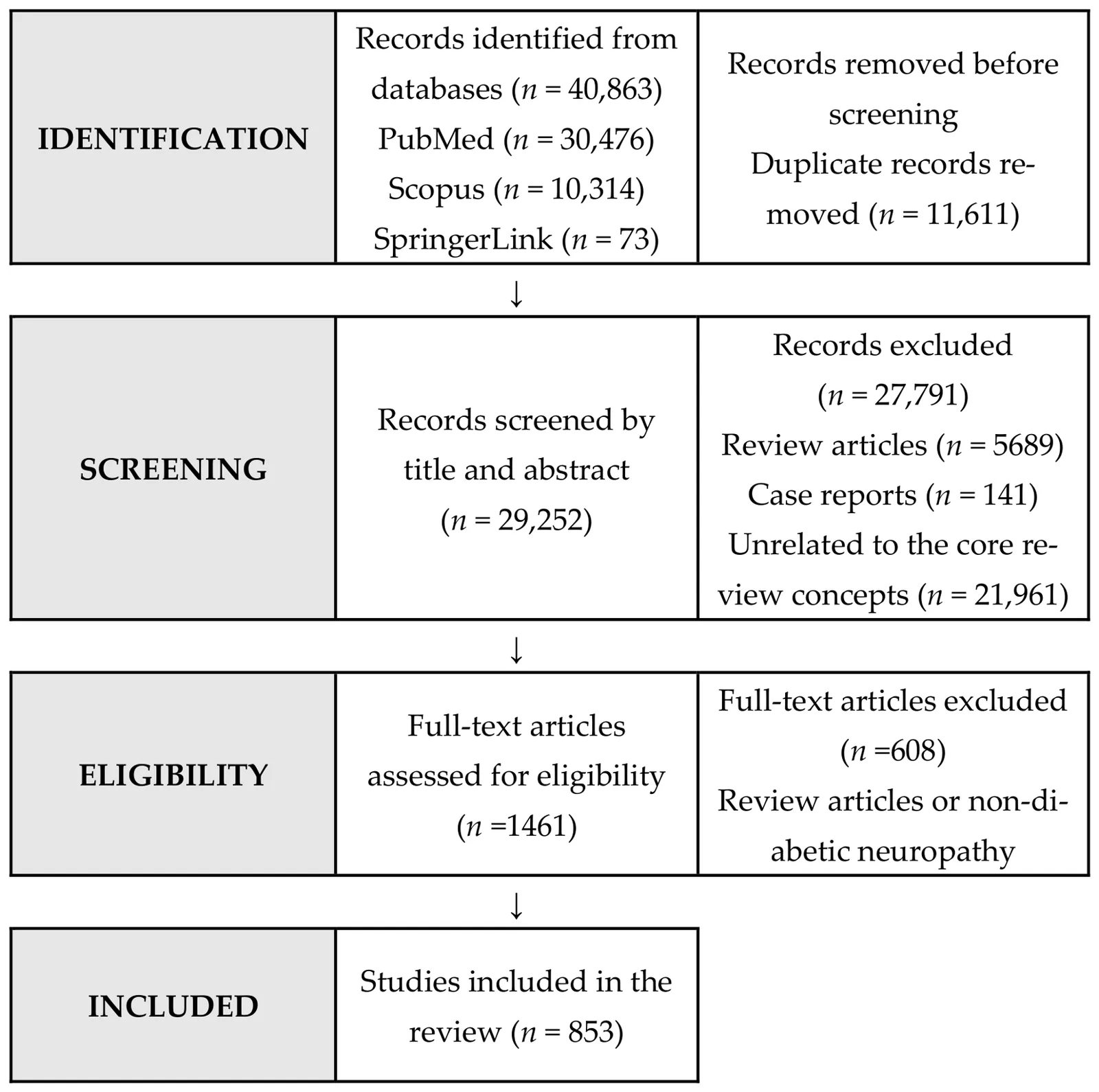
PRISMA-ScR Flow Diagram of Study Selection.

**Figure 2:**
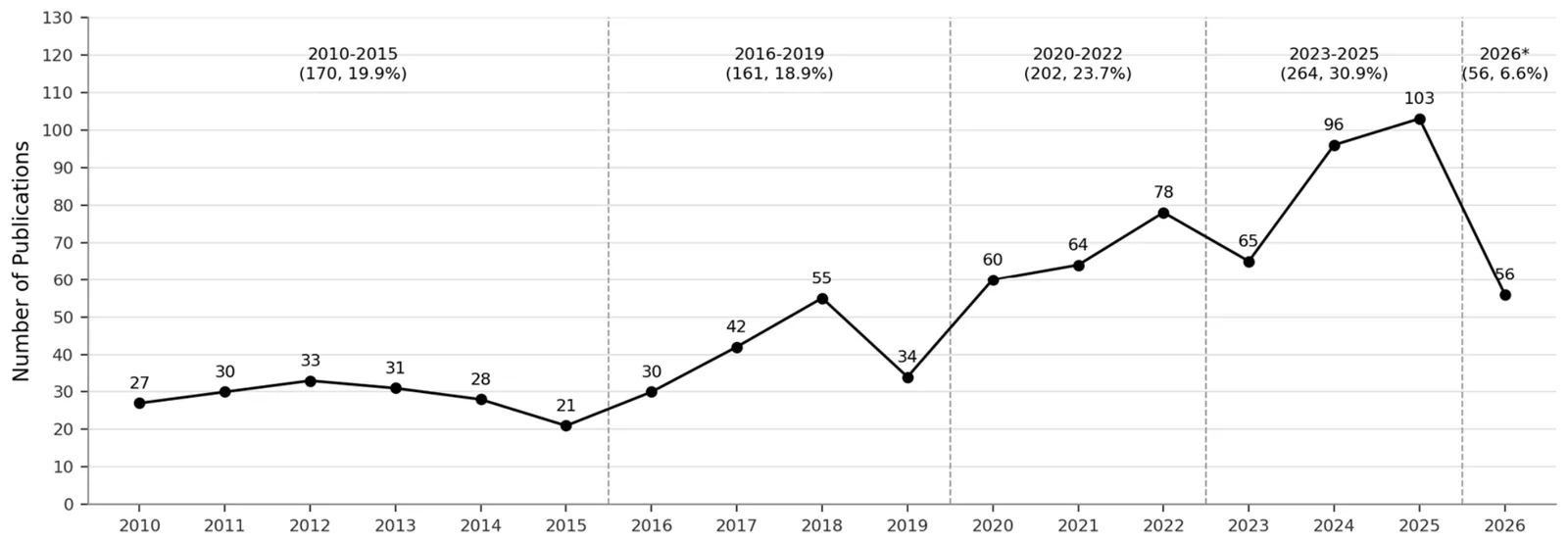
Publication Trends of Mitochondrial Dysfunction Studies in Diabetic Neuropathy. (2010–2026; *n* = 853).

**Figure 3:**
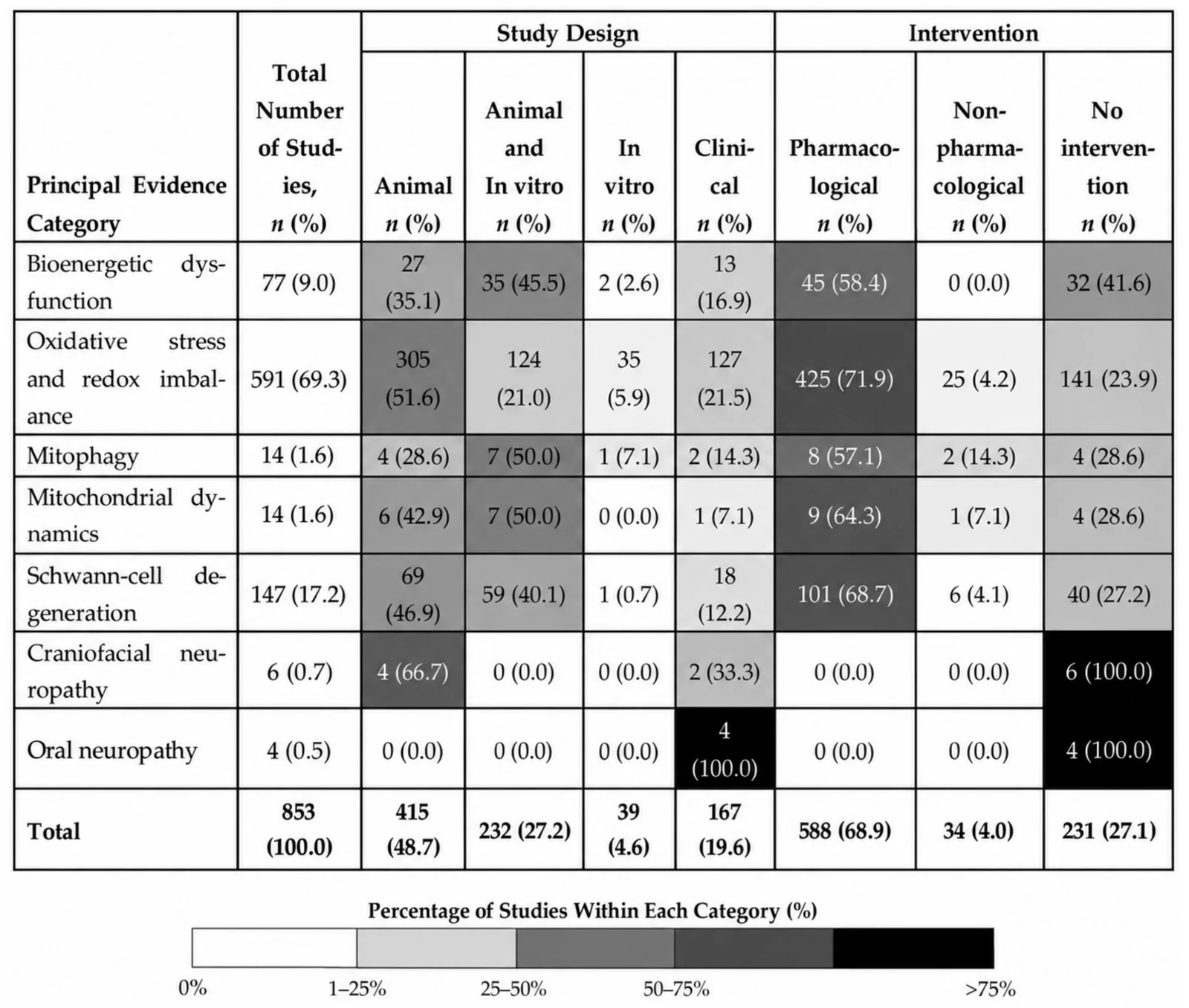
Evidence Mapping of Principal Evidence Categories in Diabetic Neuropathy (*n* = 853).

**Figure 4:**
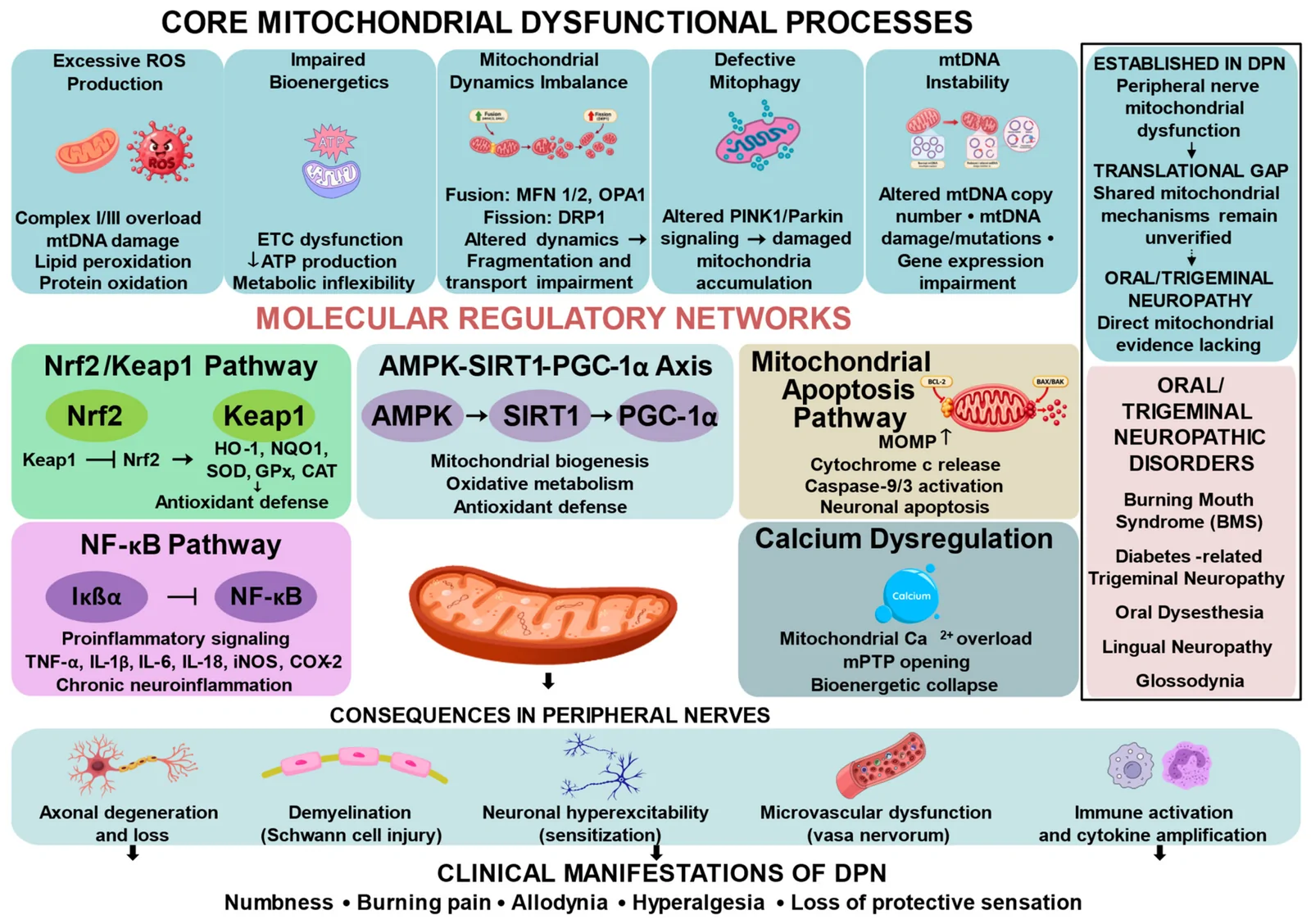
Integrated mitochondrial mechanisms in diabetic peripheral neuropathy and the translational gap toward oral and trigeminal neuropathy. In DPN, reported mitochondrial abnormalities include excessive ROS production, impaired bioenergetics, altered mitochondrial dynamics, defective mitophagy, mtDNA instability, and calcium dysregulation. These processes interact with Nrf2/Keap1-associated antioxidant defense, NF-κB-associated inflammatory signaling, AMPK-SIRT1-PGC-1α-associated mitochondrial homeostasis, and mitochondria-related apoptotic pathways, and have been associated with axonal degeneration, Schwann-cell injury, neuronal sensitization, microvascular dysfunction, and immune activation. Directional alterations shown in the figure represent findings reported across experimental and clinical studies and may vary among models and study settings; they should not be interpreted as universal features of DPN. Although mitochondrial dysfunction is increasingly characterized in peripheral DPN, comparable mechanisms remain insufficiently investigated in oral and trigeminal neuropathic disorders. The dashed translational pathway therefore represents a knowledge gap rather than an established causal link.

**Table 1:** Overview of the Sequential Search Strategy and Objectives.

Search Set	Focus	Purpose
Search Set 1	Diabetic neuropathy + mitochondrial dysfunction	To identify the core evidence on mitochondrial mechanisms in diabetic neuropathy.
Search Set 2	Experimental models of diabetic neuropathy	To identify mechanistic evidence from animal and in vitro studies.
Search Set 3	Oral/craniofacial neuropathic disorders + mitochondrial dysfunction	To identify studies examining mitochondrial dysfunction in oral and craniofacial neuropathic disorders.
Search Set 4	Diabetes-related neuropathy + oral/craniofacial neuropathic disorders	To identify translational evidence linking diabetic neuropathy with oral and craniofacial neuropathic manifestations.
Search Set 5	Diabetes + mitochondrial dysfunction + oral/craniofacial neuropathic disorders	To identify evidence integrating all three concepts and to define remaining knowledge gaps.

**Table 2:** Characteristics of the Included Studies.

Characteristic	Category	*n* (%)
Publication year	2010–2020	391 (45.8)
	2021–2026	462 (54.2)
Study design	Animal	415 (48.7)
	Animal + in vitro	232 (27.2)
	In vitro	39 (4.6)
	Clinical	167 (19.6)
Biological model	Cell culture	150 (17.6)
	STZ-induced rats	248 (29.1)
	STZ-induced mice	61 (7.2)
	High-fat diet model	53 (6.2)
	*db/db mice*	39 (4.6)
	Other animal model	135 (15.8)
	Human participants	167 (19.6)
Neuropathy phenotype	Diabetic neuropathy	551 (64.6)
	Painful DPN	248 (29.1)
	Cardiovascular autonomic neuropathy	31 (3.6)
	Craniofacial neuropathy	19 (2.2)
	Oral neuropathy	4 (0.5)

Note: The 2026 publication count includes studies identified through August 2026; therefore, 2026 represents a partial year.

**Table 3:** Evidence Mapping of Principal Evidence Categories in Diabetic Neuropathy (*n* = 853).

		Study Design				Intervention		
Principal Evidence Category	Studies, *n* (%)	Animal, *n* (%)	Animal + In Vitro, *n* (%)	In Vitro, *n* (%)	Clinical *n* (%)	Pharmacological, *n* (%)	Non-Pharmacological, *n* (%)	No Intervention, *n* (%)
Bioenergetic dysfunction	77 (9.0)	27 (35.1)	35 (45.5)	2 (2.6)	13 (16.9)	45 (58.4)	0 (0.0)	32 (41.6)
Oxidative stress and redox imbalance	591 (69.3)	305 (51.6)	124 (21.0)	35 (5.9)	127 (21.5)	425 (71.9)	25 (4.2)	141 (23.9)
Mitophagy	14 (1.6)	4 (28.6)	7 (50.0)	1 (7.1)	2 (14.3)	8 (57.1)	2 (14.3)	4 (28.6)
Mitochondrial dynamics	14 (1.6)	6 (42.9)	7 (50.0)	0 (0.0)	1 (7.1)	9 (64.3)	1 (7.1)	4 (28.6)
Schwann-cell degeneration	147 (17.2)	69 (46.9)	59 (40.1)	1 (0.7)	18 (12.2)	101 (68.7)	6 (4.1)	40 (27.2)
Craniofacial neuropathy	6 (0.7)	4 (66.7)	0 (0.0)	0 (0.0)	2 (33.3)	0 (0.0)	0 (0.0)	6 (100.0)
Oral neuropathy	4 (0.5)	0 (0.0)	0 (0.0)	0 (0.0)	4 (100.0)	0 (0.0)	0 (0.0)	4 (100.0)
Total	853 (100.0)	415 (48.7)	232 (27.2)	39 (4.6)	167 (19.6)	588 (68.9)	34 (4.0)	231 (27.1)

Note: Studies reporting more than one mechanism were classified according to their principal evidence category to avoid double counting. When multiple mechanisms were identified, categories were prioritized in the following order: oral neuropathy, craniofacial neuropathy, Schwann cell degeneration, mitochondrial dynamics, mitophagy, bioenergetic dysfunction, and oxidative stress/redox imbalance. Studies that did not meet the criteria for a higher-priority category were classified under oxidative stress/redox imbalance. Percentages for study design and intervention were calculated within each principal evidence category and may not total 100% because of rounding. Individual study assignments are provided in Supplementary Table S2.

## Data Availability

No new data were created or analyzed in this study. Data sharing is not applicable to this article.

## Supplementary Materials

The following supporting information can be downloaded at: https://www.mdpi.com/article/10.3390/ijms27198647/s1.

## Abbreviations

ACSL4	Acyl-CoA Synthetase Long-Chain Family Member 4
AGE-RAGE	Advanced Glycation End Products–Receptor for Advanced Glycation End Products
AICAR	5-Aminoimidazole-4-Carboxamide Riboside
AMPK	AMP-Activated Protein Kinase
Bax	Bcl-2-Associated X Protein
Bcl-2	B-Cell Lymphoma 2
BDNF	Brain-Derived Neurotrophic Factor
CAT	Catalase
CHOP	C/EBP Homologous Protein
c-Jun	c-Jun Proto-Oncogene
DNA	Deoxyribonucleic Acid
DPN	Diabetic Peripheral Neuropathy
GPx	Glutathione Peroxidase
GPX4	Glutathione Peroxidase 4
GRP78	Glucose-Regulated Protein 78
GSH	Glutathione
HO-1	Heme Oxygenase-1
IL-1β	Interleukin-1 Beta
IL-6	Interleukin-6
MBP	Myelin Basic Protein
MDA	Malondialdehyde
MFN2	Mitofusin 2
MPZ	Myelin Protein Zero
mTORC1	Mechanistic Target of Rapamycin Complex 1
NAD+	Nicotinamide Adenine Dinucleotide
NF-κB	Nuclear Factor Kappa B
NLRP3	NLR Family Pyrin Domain Containing 3
NQO1	NAD(P)H Quinone Dehydrogenase 1
NRF1	Nuclear Respiratory Factor 1
NRF2	Nuclear Factor Erythroid 2-Related Factor 2
PGC-1α	Peroxisome Proliferator-Activated Receptor Gamma Coactivator 1-Alpha
RNA	Ribonucleic Acid
ROS	Reactive Oxygen Species
SIRT1	Sirtuin 1
SIRT3	Sirtuin 3
SLC7A11	Solute Carrier Family 7 Member 11
SOD	Superoxide Dismutase
TNF-α	Tumor Necrosis Factor-Alpha
TRPV1	Transient Receptor Potential Vanilloid 1
